# Progressive Locomotor Ataxy

**Published:** 1881-04-20

**Authors:** T. B. Greenley

**Affiliations:** Kentucky


### PROGRESSIVE LOCOMOTOR ATAXY.

BY T. B. GREENLEY, M.D., OF KENTUCKY.

From the best observations it is demonstrated that this disease is located in the posterior column of the spinal cord, and, of course, belongs to the great family of diseases known as neuroses. Its pathology seems to be a sclerosis of that portion of the cord, and commences in the lumbar region. The patient at first may only be conscious of a slight tingling sensation or numbness in the extremities, amounting as it progresses to partial anæsthesia.

The most prominent symptom in the disease as it progresses is the want of accommodation on the part of the muscles of locomotion. The patient, on attempting to walk, shuffles along like a drunken man. The muscles refuse to obey the will, and in making the effort he may fall. Owing to partial anæsthesia, the ground feels soft, as though he was walking on cotton or wool. His eyes serve as crutches and guide him in his steps, or otherwise he is liable to fall. He, therefore, is unable to walk in the dark. The sense of touch is greatly impaired, being unable to distinguish what he may hold in his hand without looking at it.

The tendency of the disease is to grow worse, and finally to bring the patient to bed. The cause of ataxy is not, I presume, well understood, as we may see it developed in those who are apparently robust, and who may have previously enjoyed good health.

The prognosis is generally unfavorable as far as entire recovery is concerned; yet independent of this a prudent man may, with proper treatment, live many years, and enjoy comparative comfort. Fortunately it is comparatively a rare disease; I have seen but two cases in a practice of thirty-six years. Both of these were in young people of previous good health. One, a female of sixteen; the other, a young man, both unmarried. The first was not my patient, and I am unable to give a history of the case or its termination. The second, a young man twenty-eight years old, stoutly made and of usual robust health, applied to me for treatment in October last. He had six weeks before begun to feel slight numbness in the extremities, which gradually increased until he found himself unable to place his feet properly when walking. He, however, continued to work a while longer, being engaged as a hand at a saw-mill.

When I saw him he could still hobble along, frequently staggering, and sometimes nearly falling. I directed him to suspend all efforts to work, and keep comparatively quiet.

The treatment consisted in the use of tinctures of nux vomica and belladonna, of each 15 drops three times a day, with proper diet. Frictions and stimulating applications over lumbar region.

In two weeks from the time I first saw him, he was unable to walk, but his appetite and digestion continued good. This inability to walk only lasted about two weeks before he began to improve; and by the first of January he was able to walk better than when I first saw him. He continued to improve and is now apparently as well as he was previous to the commencement of the attack. He feels no difficulty whatever in commanding the use of his feet in locomotion. He seems so entirely relieved that I advised him to discontinue the medicine some time since.

The most approved treatment of ataxy seems to be the use of ergot, but in my case the patient could not take it on account of intolerance on the part of the stomach. Nitrate of silver is also highly recommanded in this trouble, but as the nux vomica and belladonna seemed to do well I did not use it.

I do not recollect having seen belladonna recommended for ataxy, but as I have had good results from it in some other affections belonging to the class neuroses, I deemed it worthy of trial in this disease. Should any member of the profession who may see this article, and who may happen to have a case of ataxy on hand, I hope he will give the treatment I used in this case a fair trial, and report the result.

